# Intrapartum factors associated with neonatal hypoxic ischemic encephalopathy: a case-controlled study

**DOI:** 10.1186/s12884-017-1610-3

**Published:** 2017-12-11

**Authors:** Vanessa E. Torbenson, Mary Catherine Tolcher, Kate M. Nesbitt, Christopher E. Colby, Sherif A. EL-Nashar, Bobbie S. Gostout, Amy L. Weaver, Michaela E. Mc Gree, Abimbola O. Famuyide

**Affiliations:** 1 Department of Obstetrics and Gynecology, Mayo Clinic Rochester, 200 First Street SW, Rochester, MN 55905 USA; 2 Department of Pediatric and Adolescent Medicine, Mayo Clinic Rochester, Rochester, MN USA; 3 Division of Female Pelvic Medicine and Reconstructive Surgery, University Hospitals, Cleveland, OH USA; 4 Division of Biomedical Statistics and Informatics, Mayo Clinic Rochester, Rochester, MN USA

**Keywords:** Hypoxic ischemic encephalopathy, Neonatal encephalopathy, Intrapartum factors, Risk factors

## Abstract

**Background:**

Neonatal encephalopathy (NE) affects 2–4/1000 live births with outcomes ranging from negligible neurological deficits to severe neuromuscular dysfunction, cerebral palsy and death. Hypoxic ischemic encephalopathy (HIE) is the sub cohort of NE that appears to be driven by intrapartum events. Our objective was to identify antepartum and intrapartum factors associated with the development of neonatal HIE.

**Methods:**

Hospital databases were searched using relevant diagnosis codes to identify infants with neonatal encephalopathy. Cases were infants with encephalopathy and evidence of intrapartum hypoxia. For each hypoxic ischemic encephalopathy case, four controls were randomly selected from all deliveries that occurred within 6 months of the case.

**Results:**

Twenty-six cases met criteria for hypoxic ischemic encephalopathy between 2002 and 2014. In multivariate analysis, meconium-stained amniotic fluid (aOR 12.4, 95% CI 2.1–144.8, *p* = 0.002), prolonged second stage of labor (aOR 9.5, 95% CI 1.0–135.3, *p* = 0.042), and the occurrence of a sentinel or acute event (aOR 74.9, 95% CI 11.9-infinity, *p* < 0.001) were significantly associated with hypoxic ischemic encephalopathy. The presence of a category 3 fetal heart rate tracing in any of the four 15-min segments during the hour prior to delivery (28.0% versus 4.0%, *p* = 0.002) was more common among hypoxic ischemic encephalopathy cases.

**Conclusion:**

Prolonged second stage of labor and the presence of meconium-stained amniotic fluid are risk factors for the development of HIE. Close scrutiny should be paid to labors that develop these features especially in the presence of an abnormal fetal heart tracing. Acute events also account for a substantial number of HIE cases and health systems should develop programs that can optimize the response to these emergencies.

## Background

Hypoxic ischemic encephalopathy (HIE) is a subset of neonatal encephalopathy (NE) attributable to intrapartum events leading to acute hypoxia-ischemia. A recent executive summary from the American College of Obstetricians and Gynecologists (ACOG) and American Academy of Pediatrics (AAP) Joint Task Force on Neonatal Encephalopathy outlines the need for more research on the incidences of both NE and HIE [1]. Imprecise definitions to distinguish HIE from NE have made determining causal factors for HIE difficult.

Previous studies have examined antepartum and intrapartum events that are associated with neonatal HIE [2–5]. Martinez-Biarge et al. reported seven intrapartum factors and one antepartum factor associated with the development of HIE—prolonged rupture of membranes, abnormal cardiotocography, thick meconium, sentinel event, shoulder dystocia, tight nuchal cord, failed vacuum, and gestational age ≥ 41 weeks [3]. In their case-control study of infants born beyond 36 weeks gestation, Hayes et al. identified several risk factors for the development of HIE including thick meconium, fetal growth restriction, large head circumference, oligohydramnios, male fetal sex, fetal bradycardia, maternal pyrexia, and increased uterine contractility [6].

Identifying risk factors for HIE are key, because if they develop intrapartum and are modifiable, specific interventions or labor and delivery practices may impact the incidence of HIE. To date, studies to reduce the incidence of HIE have yielded conflicting results. In one study, introduction of a bundle of interventions including practice standardization and training in fetal heart rate (FHR) tracing interpretation did not affect the proportion of neonates with umbilical artery pH < 7.20 [7]. In contrast, Draycott et al. showed a 50% reduction in HIE cases from 27.3 to 13.6 per 10,000 births and a 50% decrease in infants born with a 5-min Apgar score < 6 following the introduction of obstetric emergency training courses at a tertiary hospital in the United Kingdom [8]. This study suggests that obstetrics training and intervention may decrease the incidence of HIE.

The objectives of this study are to determine the risk factors for HIE in infants delivered at our institution using a strict criteria for case selection that includes brain imaging when available to exclude encephalopathy that is not a result of intrapartum events. We hope to identify factors that are actionable that can alter the development of HIE.

## Methods

This is a case-control study of all live born singleton infants delivered at ≥ 35 weeks of gestation at Mayo Clinic Rochester between January 1, 2002 and October 1, 2014. Infants with major congenital malformations or known genetic defects were excluded. Deliveries were also excluded if research authorization was declined for either mother or infant in accordance with Minnesota state law. This study was approved by the Mayo Clinic Institutional Review Board.

Cases were identified by searching the electronic medical record (EMR) for The International Classification of Diseases, 9th Revision (ICD-9) codes (768.0–768.9) for “intrauterine hypoxia” and “birth asphyxia” to identify infants at risk of the development of NE and HIE. In addition, departmental obstetric and neonatal databases were searched for potential cases missed by the ICD-9 search. Finally, procedure codes for magnetic resonance imaging (MRI) were used to identify any infant who had an MRI performed.

Once potential cases were identified, individual EMRs were manually reviewed. Cases first met criteria for NE as follows: infants born at ≥ 35 0/7 weeks of gestation with disturbed neurological function manifested by subnormal level of consciousness, difficult initiating or maintaining respiration, abnormal tone and reflexes, or seizures. Infants with NE were identified as HIE cases if they met the following criteria: Apgar score < 5 at 5 min and/or umbilical cord artery acidemia (pH < 7.1 or base excess ≥ 12 mmol/L) and/or neuroimaging evidence of acute brain injury based on MRI or computed tomography (CT). Radiographic data were extracted from either MRI or CT reports performed within 1 week of birth. MRI reports were reviewed for the presence of abnormal signal intensity in the basal ganglia and thalami, loss of normal signal intensity in the posterior limb of the internal capsule, or acutely evolving focal infarction in an arterial territory or in a parasagittal or watershed distribution [9]. CT reports were examined for brain swelling, cortical highlighting, focal or global loss of gray/white matter differentiation, and hypoattenuation [9]. CT scans were utilized only if MRI was not available as was the case with deliveries in the early 2000s.

For each HIE case, four controls were randomly selected from all deliveries that occurred within 6 months of the case delivery and matched for gestational age (± 1 week) and maternal age (± 2 years). Maternal and obstetric history and delivery details were abstracted from the EMR.

Prolonged second stage of labor was defined as greater than 2 h in a multiparous patient and greater than 3 h in a primiparous patient without regional anesthesia. For those with regional anesthesia, a second stage greater than 3 h in a multiparous patient and greater than 4 h in a primiparous patient was used.

The diagnonisis of chorioamnionitis was directly abstracted from the labor record and was assigned if the patient developed at temprature of ≥ 38.0C and was treated with intravenous antibiotics.

FHR tracings were retrieved electronically from fetal monitoring archives. Tracings from the first hour after admission and the hour prior to delivery were reviewed. Two obstetricians (AOF and MCT), blinded to the patient’s identity and outcome, retrospectively interpreted each tracing in 15-min segments and assigned each segment a category (1–3, or) using the Eunice Kennedy Shriver National Institutes of Child Health and Human Development (NICHD) criteria [10]. A third obstetrician (VET) independently interpreted the segments for which there was disagreement and assigned the consensus category used for the final analysis. The predominant category for each tracing for the first and final hours was determined from the consensus ratings. If less than 1 h of the FHR tracing was available, then this was analyzed as the hour before delivery. If a tracing had a 15-min segment that could not be read, it was categorized as uninterpretable.

Maternal and infant baseline characteristics were compared between cases and controls using the chi-square or Fishers’ exact test for categorical variables and the Wilcoxon rank-sum test or two sample t-tests for continuous variables. Conditional logistic regression models were used to estimate the odds ratio (OR) and corresponding 95% confidence intervals (CI) and *p*-value for each risk factor of interest. Multivariable exact conditional logistic regression models were fit considering all variables with *p* < 0.20 based on univariate analysis and first examining all one-variable models, all two-variable models, and all three-variable models. Variables were retained in the final multivariable model if *p* < 0.05. Non-applicable responses (e.g. those who did not labor for the determination of prolonged second stage) were coded with dummy variables in order to include all patients in the analyses. A weighted kappa statistic was used to quantify the agreement between the FHR tracing interpretations by the two obstetricians. The distribution of the predominant FHR tracing category (based on consensus) was compared between HIE cases and controls using the Wilcoxon rank sum test. All calculated *p*-values were two-sided and p-values less than 0.05 were considered statistically significant. Statistical analysis was performed using the SAS version 9·3 software package (SAS Institute, Inc.; Cary, NC).

## Results

**Fig. 1:**
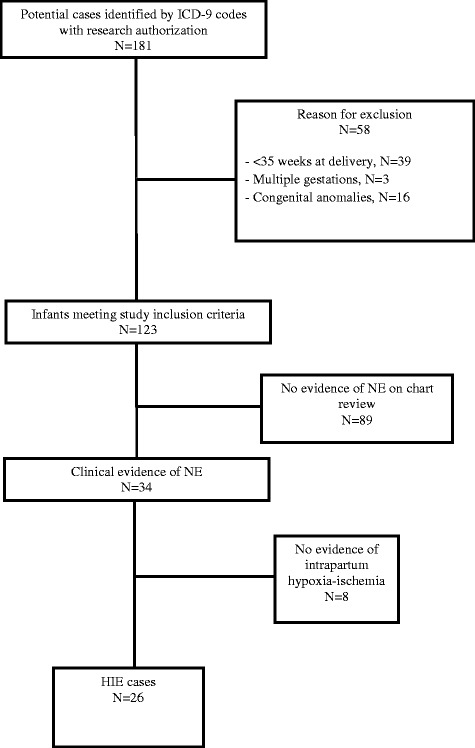
Flow diagram of hypoxic ischemic encephalopathy case selection

Twenty-six cases met criteria for HIE (Fig. 1). Baseline characteristics show that our study population is predominately young, white and privately insured. While not reaching statistical significance, self-identified black race was more common among cases than controls (19.2% vs 3.8%, *p* = .08) (Table 1). Among the HIE cases, over half (53.9%) had a cesarean delivery after a course of labor, 19.2% had a cesarean delivery without laboring, 23.1% had a spontaneous vaginal delivery and 3.8% had an operative vaginal delivery. In comparison, the majority (76.0%) of the controls had a spontaneous vaginal delivery, 10.6% had a cesarean delivery after a course of labor,7.6% had an operative vaginal delivery, and 5.8% had a cesarean delivery without laboring.

**Table 1:** Maternal and neonatal characteristics of cases and controls

Characteristics	Cases (*N* = 26)	Controls (*N* = 104)	*P* ^a^
Maternal			
Age (years), mean (SD)	26.7 (3.8)	26.6 (3.7)	0.97
BMI (kg/m^2^), mean (SD)	25.7 (5.8)	26.1 (6.0)	0.77
Medicaid insurance, N (%)	6 (23.1%)	34 (32.7%)	0.34
Race, N (%)			0.08
Caucasian	18 (69.2)	89 (85.6)	
Black or African American	5 (19.2)	4 (3.8)	
Asian	2 (7.7)	6 (5.8)	
Other	1 (3.8)	3 (2.9)	
Unknown	0 (0.0)	2 (1.9)	
Nulliparous, N (%)	14 (53.8)	41 (39.4)	0.18
Neonatal			
Male sex, N (%)	18 (69.2)	63 (60.6)	0.42
Gestational age (weeks), median (IQR)	39 (38, 40)	39 (38, 40)	0.94
Birth weight (grams), mean (SD)	3514 (596)	3448 (530)	0.58

*Abbreviations*: *BMI* body mass index, *IQR* interquartile range, *SD* standard deviation

^a^Comparisons evaluated using the chi-square for sex, nulliparous and Medicaid insurance, the Wilcoxon rank-sum test for gestational age, the two-sample t-test for birth weight, maternal age and BMI, and the Fisher’s exact test for maternal race

Potential risk factors for HIE were evaluated in univariate analysis (Table 2). When cases were compared to controls, cases were more likely to have presence of meconium-stained amniotic fluid (42.3% vs. 15.4%, *p* = 0.004), prolonged second stage of labor (29.4% vs. 4.4%, *p* = 0.007), a sentinel or acute event compared to controls (23.1% vs. 0%, *p* < 0.001), and the presence of at least one category 3 segment in any of the four 15-min segments in the last hour before delivery (28.0% vs. 4.0%, *p* = 0.002). Rates of fetal growth restriction, chorioamnionitis and oxytocin use were similar among cases and controls.

**Table 2:** Clinical and labor characteristics evaluated as risk factors for hypoxic ischemic encephalopathy

Characteristic	Cases (*N* = 26) N (%)	Controls (*N* = 104) N (%)	Univariate analysis		Multivariable analysis^g^	
			Unadjusted OR (95% CI)	*P*	Adjusted OR (95% CI)	*P*
Prior cesarean delivery	6 (23.1%)	11 (10.6%)	2.53 (0.83, 7.75)	0.10		
Chronic hypertension	2 (7.7%)	1 (1.0%)	8.00 (0.72, 88.23)	0.09		
Gestational diabetes	1 (3.8%)	4 (3.8%)	1.00 (0.02, 10.11)	1.00		
Fetal growth restriction	0 (0%)	2 (1.9%)	1.66 (0, 13.89)	1.00		
Oligohydramnios	1 (3.8%)	2 (1.9%)	2.00 (0.18, 22.06)	0.57		
Preterm labor	2 (7.7%)	4 (3.8%)	2.67 (0.33, 21.54)	0.36		
Meconium-stained amniotic fluid^a^	11 (42.3%)	16 (15.4%)	4.62 (1.63, 13.06)	0.004	12.41 (2.10, 144.83)	0.002
Chorioamnionitis	2 (7.7%)	3 (2.9%)	3.15 (0.43, 23.36)	0.26		
Oxytocin use	14 (53.8%)	50 (48.1%)	1.26 (0.53, 3.00)	0.60		
Abnormal first stage of labor^b,c^	12/17 (70.6%)	48/94 (51.1%)	2.19 (0.71, 6.76)	0.17		
Prolonged second stage of labor^d^	5/17 (29.4%)	4/91 (4.4%)	11.24 (1.92, 65.58)	0.007	9.49 (1.06, 135.30)	0.042
Any sentinel or acute event^e^	6 (23.1%)	0 (0%)	32.66 (6.18, infinity)	< 0.001	74.86 (11.86, infinity)	< 0.001
Any ‘category 3’ segment in the last hour of labor^f^	7/25 (28.0%)	4/101 (4.0%)	7.30 (2.11, 25.30)	0.002		

^a^Meconium-stained amniotic fluid present either before or after 6 cm of dilation

^b^The first stage of labor could not be classified for 9 HIE cases and 10 controls because they either have a cesarean section without laboring (*N* = 5 cases and *N* = 6 controls) or they first presented at 10 cm (N = 2 controls) or they never reached an active phase before proceeding to a cesarean section (N = 1 case and N = 1 control) or they only had 1 measurement in the active phase before proceeding to a cesarean section (*N* = 3 case and *N* = 1 control)

^c^Abnormal first stage of labor included patients with protracted labor (cervical dilation <1.2 cm per hour), secondary arrest (lack of cervical dilation for >2 h after patient reached 7 cm) and combined disorders (patients with both protracted labor and secondary arrest)

^d^The second stage of labor could not be classified for 9 HIE cases and 13 controls because they either have a cesarean section without laboring (*N* = 5 cases and *N* = 6 controls) or they did not reach 10 cm before proceeding to a cesarean section (*N* = 4 cases and *N* = 7 controls)

^e^A sentinel event was defined as an acute event that emergently altered the course of labor; these included uterine rupture, cord prolapse, placental abruption, fetal exsanguination/vasa previa, amniotic fluid embolism, and maternal collapse

^f^One HIE case and 3 controls had insufficient information for categorizing the fetal heart tracings

^g^Multivariable exact conditional logistic regression models were fit considering all variables with *p* < 0.20 based on univariate analysis and first examining all one-variable models, all two-variable models, and all three-variable models. Variables were retained in the final multivariable model if *p* < 0.05

Based on multivariable logistic regression analysis, meconium-stained amniotic fluid (aOR 12.4, *p* = 0.002), prolonged second stage (aOR 9.5, *p* = 0.04), and occurrence of a sentinel or acute event (aOR 74.9, *p* < 0.001) were significantly associated with a higher likelihood of HIE (Table 2). Among the 26 HIE cases, 20 (76.9%) had at least one of these three factors present (two cases each had two factors and 18 cases had 1 factor) compared to 18 (17.3%) of the 104 controls (two controls each had two factors and 16 controls had one factor).

Interobserver agreement in the interpretation of FHR tracings was fair to moderate, with an overall weighted kappa statistic of 0.64 based on all segments combined. The distribution of the predominant category for each tracing during the first hour after admission was not significantly different between HIE cases and controls (*p* = 0.20), however, the predominant category rating was higher for the HIE cases compared to controls during the last hour prior to delivery (*p* = 0.02) (Table 3). FHR tracings for both cases and controls tended to progress from category 1 to category 2 from admission to delivery.

**Table 3:** Comparison of the fetal heart rate tracing categorizations during the first hour after admission and last hour prior to delivery

Predominant category during the first hour of admission, N (%)^a^	Cases (*N* = 24)^b^	Controls (*N* = 100)^c^	*P*-value^f^
			0.20
Category 1	14 (58.3%)	71 (71.0%)	
Category 1 or 2	2 (8.3%)	6 (5.8%)	
Category 2	7 (29.2%)	2 (23.1%)	
Category 2 or 3	1 (4.2%)	0	
Category 3	0	0	
Predominant category during the last hour prior to delivery, N (%)^a^	Cases (*N* = 25)^d^	Controls (*N* = 101)^e^	*P*-value^f^
			0.02
Category 1	1 (4.0%)	8 (7.9%)	
Category 1 or 2	1 (4.0%)	11 (10.9%)	
Category 2	20 (80.0%)	82 (81.2%)	
Category 2 or 3	2 (8.0%)	0	
Category 3	1 (4.0%)	0	

*Abbreviation*: *FHR* fetal heart rate tracings

^a^For each patient the predominant category was defined as the most common rating among the four 15-min segments from the FHR tracings using the consensus ratings

^b^Of the 26 HIE cases, 24 had FHR tracings available during the first hour after admission (20 had 4 interpretable segments, 3 had 3 interpretable segments, and 1 had 1 interpretable segment)

^c^Of the 104 controls, 100 had FHR tracings available during the first hour after admission (80 had 4 interpretable segments, 11 had 3 interpretable segments, 5 had 2 interpretable segments, and 4 had 1 interpretable segment)

^d^Of the 26 HIE cases, 25 had FHR tracings available during the last hour prior to delivery (23 had 4 interpretable segments, 1 had 3 interpretable segments, and 1 had 1 interpretable segment)

^e^Of the 104 controls, 101 had FHR tracings available during the last hour prior to delivery (85 had 4 interpretable segments, 9 had 3 interpretable segments, 4 had 2 interpretable segments, and 3 had 1 interpretable segment)

^f^ *P*-value based on the Wilcoxon rank-sum test taking into account the ordinal nature of the predominant category

Among the 26 HIE cases, six had sentinel or acute event which included placental abruption, uterine rupture and shoulder dystocia. Of the six HIE cases with a sentinel event, one had therapeutic cooling and two died. Brain imaging was performed in four cases; two MRI and two CT scans: two cases had no imaging documented, the MRI’s obtained detailed evidence of hypoxic-ischemia. Among the 20 HIE cases without a sentinel event, seven had therapeutic cooling, three died and the six of nine MRI’s showed clear evidence of ischemia.

## Discussion

We identified three intrapartum risk factors for HIE including the presence of meconium-stained amniotic fluid, prolonged second stage of labor, and occurrence of a sentinel event. In addition, HIE cases were more likely to have at least one 15-min segment of FHR tracing categorized as abnormal in the hour preceding delivery. Baseline demographic data show a trend towards overrepresentation of black race among the cases, although this did not reach the level of significance. This interesting finding was not observed in the private vs Medicaid insurance categorization- as a surrogate for socioeconomic status. Larger studies will be needed to evaluate any role race plays in the occurrence of HIE.

The significance of meconium-stained amniotic fluid is controversial and not universally accepted as a cause for concern. Several studies have found no association between the presence of meconium and low Apgar scores or hypoxia [11, 12]. However, other studies have found a correlation between passage of thick meconium and poor obstetrical outcomes [13]. Specifically, Hayes et al. recently found that higher grade meconium was associated with HIE [6]. Our results show that meconium passage was increased among HIE cases, suggesting that its presence may indicate low fetal reserve for further stressors.

Our study confirms the lack of predictability of FHR tracings for HIE, consistent with a recent report that found no differences in FHR tracing categorization in a cohort of newborns who underwent whole body cooling for HIE [14]. Although both cases and controls had a higher percentage of category 2 tracing as labor progressed, the current NICHD classification does not indicate which (if any) category 2 tracings require immediate delivery versus continued intrauterine resuscitation and expectant management. Nevertheless, we found more frequent occurrence of category 3 tracing in any segment in the last hour prior to delivery in HIE cases.

We found a significant association of prolonged second stage and HIE, which is consistent with other recent cohort studies that demonstrated an association between prolonged second stage of labor and adverse neonatal outcomes (low Apgar scores, neonatal intensive care unit admission, and increased composite neonatal morbidity) [15–17]. This is in contrast to two large cohort studies that did not find increased neonatal morbidity with prolonged second stage of labor, although these studies did not address HIE specifically [14, 15]. Interestingly, four of the 26 cases had cesarean deliveries complicated by a difficult fetal head extraction which is more likely to occur after a prolonged labor. Thus the association of a prolonged second stage and HIE may be multifactorial.

Finally, we found that obstetric sentinel events are associated with HIE. HIE cases stemming from a sentinel event were more likely to result in death of the neonate. Although sentinel events may be unavoidable, prompt recognition and response to these events may impact neonatal outcomes. Draycott and colleagues showed a reduction in low 5-min Apgar scores and HIE rates with the introduction of obstetric emergency training [8]. Furthermore, team training at a large tertiary hospital in the UK was associated with a reduction of low Apgar scores and admission to the neonatal intensive care unit NICU in cases of cord prolapse [18]. These studies indicate that outcomes for unpreventable emergencies can be improved with simulation or team-based training.

Our study strengths include a matched case-control methodology that allows for determination of variables impacting a relatively uncommon outcome. In addition, the cases and controls came from a single institution, minimizing differences in outcome assessment and limiting information bias. Furthermore fetal tracings were available for a majority of our cases allowing for secondary objective review. Limitations of our study include utilizing diagnosis codes to identify cases; although we may have missed cases with this method, this was minimized by the review of obstetric and neonatal databases. Another limitation was the small number of cases identified. This may have impacted our ability to establish an association between HIE and other factors such as abnormal labor. Examining the association of total length of the first stage of labor in hours rather than normal versus abnormal may have shown a linear relationship with the risk of HIE.

## Conclusions

We found a significant association between HIE and meconium-stained amniotic fluid, prolonged second stage of labor, and category 3 FHR tracing. It is important to pay close attention to these intrapartum factors while managing a labor as this may alter the course and modify the risk of HIE. However, nearly one quarter of HIE occurs acutely and may not be avoidable. It is imperative, therefore to optimize the response to emergencies as this may succeed in minimizing the incidence of HIE.

## Abbreviations

AAP: American Academy of Pediatrics
ACOG: American College of Obstetricians and Gynecologists
CT: Computed tomography
EMR: Electronic medical record
FHR: Fetal heart rate
HIE: Hypoxic ischemic encephalopathy
ICD-9: The International Classification of Diseases, 9th Revision
MRI: Magnetic resonance imaging
NE: Neonatal encephalopathy
NICHD: National Institutes of Child Health and Human Development
